# Upregulated expression of Notch1/4 - JAG-1/DLL-1 detected in allergic rhinitis

**DOI:** 10.1186/s13223-023-00793-4

**Published:** 2023-05-14

**Authors:** Eric Hjalmarsson, Marianne Petro, Susanna Kumlien Georén, Ola Winqvist, Lars Olaf Cardell

**Affiliations:** 1 Division of ENT Diseases, Department of Clinical Science, Intervention and Technology, Karolinska Institutet, Stockholm, Sweden; 2 ABClabs, Biomedicum, Stockholm, Sweden; 3 Department of Otorhinolaryngology, Karolinska University Hospital, Stockholm, Sweden

**Keywords:** Allergic rhinitis, Epithelial cells, Neutrophils, Notch, T-cells

## Abstract

**Background:**

Allergic rhinitis (AR) is a chronic disease with high prevalence. There are currently many treatments available. However, despite an often good therapeutic response, many patients still report impairment in quality of life (QoL) during the pollen season. A skewed T helper (Th)2 polarization is a well-acknowledged pathologic feature of AR. In animal models, local notch signaling in peripheral tissue seems crucial for Th2 cell differentiation and the development of AR. However, the involvement of Notch signaling in Th2 cell differentiation and the development of AR in humans remains unknown. Hence, the present study investigated the human expression of Notch receptors on CD4^+^ T-cells in nasal mucosa and blood. Correspondingly Notch ligand expression was assessed on nasal epithelial cells and neutrophils.

**Methodology:**

Nasal brush and blood samples from 18 patients with pollen-induced AR and 22 healthy controls were collected outside the pollen season. Notch 1–4 and Jagged-1,2 and Delta-like ligand 1,3–4 was analyzed using flow cytometry.

**Results:**

The fraction of CD4^+^Notch1^+^ and CD4^+^Notch4^+^ T-cells was higher in AR patients than in healthy control patients. Further, the expression levels of the Notch ligands JAG-1 and DLL-1 were increased in nasal epithelial cells from AR patients compared to healthy control patients. In addition, AR patients displayed higher expression of JAG-1 on neutrophils both in the nasal mucosa and in peripheral blood.

**Conclusion:**

The present study is the first to demonstrate increased activity in the Notch1/4 - JAG-1/DLL-1 pathways among allergic individuals. Further propagating the importance of Notch signalling in AR and blocking JAG-1 and DLL-1-induced Notch signalling by nasal epithelial cells and Neutrophils are potential targets to reduce allergic airway inflammation.

**Supplementary Information:**

The online version contains supplementary material available at 10.1186/s13223-023-00793-4.

## Introduction

Allergic rhinitis (AR) is a chronic condition with up to 30% prevalence in the Western world [1, 2]. AR can have a huge impact on quality of life (QoL) [1, 3–5]. There are currently many treatments available with antihistamines, leukotriene receptor agonists, intra-nasal steroids and oral steroids [5]. However, despite an often good therapeutic response, many patients still report impairment in QoL during the pollen season [1, 6]. The impairment in QoL leads to a loss in productivity, which results in substantial AR-related social costs [7]. Hence, there is an urge to explore new disease mechanisms that can be used to develop more effective pharmacological treatments against allergic airway inflammation [8]. A skewed T helper (Th)2 polarization is a canonic pathologic feature of AR. It promotes the production of IgE that binds to high-affinity receptors on the surface of mast cells, causing sensitization. Re-exposure to specific antigens activates the sensitized mast cells, releasing chemical mediators to evoke classic rhinitis symptoms like runny or stuffy nose, sneezing, and sometimes excessive fatigue [9].

The activation and generation of CD4^+^ Th2 cells occur in regional lymph nodes and locally in the mucosa in the presence of IL-4 [10] and through the engagement of co-stimulatory receptors [11]. Th2^+^ cells then promote the progression of AR by expressing CD40 ligand, CD28, and secretion of IL-4, and IL-13, supporting B-cells to undergo immunoglobulin class switching to produce allergen-specific IgE antibodies [12].

In the development of Th2^+^ cells, the Notch signalling pathways have been put forward as an additive factor. Local Notch-mediated signalling allows for rapid alteration of gene expression in peripheral tissue, directly modulating the T-cell response initiated in mucosa-draining lymph nodes [13, 14]. Notch signalling as a target to treat AR have been introduced earlier, and new data support this concept and also introduce possible mechanisms [15]. In mammals, this pathway consists of five ligands (Jagged (JAG) 1 and 2; delta-like ligand (DLL) 1, 3, and 4) and four receptors (Notch 1–4) [16]. Studies support that JAG-1, JAG2, Notch1, and Notch2 interaction initiates Th2 cell differentiation, and DLL-1, DLL-4, and Notch3 promote T helper 1 (Th1) cell differentiation [17]. In animal models of AR, JAG-1-Notch1, and JAG-1-Notch4, signalling has been demonstrated to induce and sustain GATA-3 expression, which polarizes T-cell responses towards Th2, promoting an allergic inflammation [18, 19]. The JAG-1-Notch1 pathway is also known to inhibit the expression of FOXP3 and the development of regulatory T-cells, further promoting the progression of allergic inflammation [20, 21]. Animal models also support that Notch signalling lowers the threshold for activation and optimizes rather than polarizes immune responses [17]. In humans, Notch signalling is less studied. Nevertheless, studies indicate that memory CD4^+^ T-cells expressing Notch1 and Notch2 are increased in patients with allergic asthma [22]. Furthermore, JAG-1 is increased in the blood of AR patients, and the levels seem to correlate with symptom severity [20]. These studies highlight the potential role of notch signalling in AR inflammation. Hence, the present study was designed to investigate the expression of Notch receptors on CD4^+^ T-cells derived from nasal mucosa and blood and the presence of their corresponding ligands on nasal epithelial cells and neutrophils derived from nasal mucosa and blood.

## Materials and methods

### Ethics Statement

The local Ethical Committee approved the study in Stockholm, Sweden (2016/823 − 31/2). All participants gave their written, informed consent. All procedures were conducted according to the principles expressed in the Declaration of Helsinki.

### Study design and patients’ characteristics

The study included 34 subjects. Sixteen patients with moderate to severe birch and grass pollen-induced seasonal AR and 18 healthy controls (HC). All participants were investigated and sampled outside the pollen season. The diagnosis of birch or grass pollen-induced AR was based on a positive history of seasonal AR and the presence of circulating allergen-specific IgE antibodies. Patients who did not meet both criteria were excluded from the study. Other exclusion criteria included a history of upper airway infection within two weeks before the visit, treatment with local or systemic corticosteroids within two months before the visit, antihistamines the day before the visit, and a history of chronic rhinosinusitis with or without the presence of nasal polyposis. ImmunoCap Rapid (Asthma/Rhinitis Adult, (Thermo Fisher Scientific, Waltham, Massachusetts, USA) was used to test for allergen-specific IgE positivity. This test detects the presence of circulating IgE to 10 common airborne allergens, including pollen (birch, timothy, mugwort, olive, wall pellitory), house dust mites (HDM), mold, and common animal allergens (cat, dog, cockroach). Patient characteristics are presented in Supplementary Table 1.

### Collection of nasal mucosal cells and blood

As previously described, sample collection from nasal mucosa was performed by brushing the nasal tissue [23]. Cells obtained were stored in Keratinocyte Serum-Free Medium (KSFM) (Gibco, Paisley, UK) at 4 °C. Blood samples (~ 9 ml) were collected in lithium heparin tubes (BD Biosciences, San José, CA, USA) and stored at room temperature.

### Flow cytometry sample preparation and acquisition

We used fluorochrome-conjugated antibodies to detect cells and Notch pathway proteins. BD Biosciences; EpCAM PerCP-5.5 (clone:EBA-1), CD45 PE-CF594 (clone: HI30), CD4 PerCP-5.5 (clone: SK3), CD5 APC (clone: UCHT2) CD3 BV711 (clone:UCHT1), CD25 BV711 (clone: 2A3) CD127 PE (clone: HIL-7R-M21) CRTH2 PE (clone: BM16) CCR5 PE-CF594 (clone: 2D7), CD16 PB (clone: 3G8), CD15 BV711 (clone:W6D3), Notch 1 BB515 (clone: MHN1-519), Notch 2 PE (clone: MHN2-25), Notch 3 BV711 (clone:MHN3-21), Notch 4 BV421 (clone:MHN4-2), JAG-1 PE (clone: MHJ1-152), Miltenyi Biotec Bergisch Gladbach, Germany; JAG-2 APC Vio770 (clone: REA1036), DLL-1 PE Vio770 (clone: MHD1-314), DLL-4 APC (MHD4-46). Before antibody staining, the cells on the nasal brush were rinsed out in PBS, and the cell suspension was passed through a 70 μm filter (BD Biosciences), centrifuged, and then the cell pellet was resuspended in PBS (GIBCO). The blood was lysed in ammonium chloride solution and sequentially washed two times with PBS to analyze neutrophils in the blood. For analysis of T-cells in the blood, PBMC was purified with SepMate tubes according to the protocol from the manufacturer. To reduce unspecific binding, all samples were pre-incubated with Fc-block (BD Biosciences). The cell suspension was then incubated with the appropriate panel of antibodies (Supplementary Table 2). Samples were stained with a full antibody panel and fluorescence minus one control. After staining, samples were washed and fixed with 1% formaldehyde (Histolab Products, Askim, Sweden) in PBS (GIBCO) and stored at four °C before sample acquisitions. Samples were acquired on an LRS Fortessa (BD Biosciences), and the performance was checked daily with a cytometer and tracking beads (CS&T) (BD Biosciences). PMT settings suggested by CS&T were used for the experiment setup. We used single stained BD comp beads (BD Biosciences) and MACS Comp Bead Kit, anti-REA (Miltenyi Biotec) for compensation. To compare median fluorescence intensity, every sample was normalized with fluorescence minus one control. A minimum of 200 T-cells or epithelial cells were analyzed for analysis of the nasal mucosa cells. Data were processed using FlowJo software© version 10.6.1 (Tree Star, Inc., Ashland, USA).

### Statistical analysis

Patient characteristics were analyzed with descriptive statistics. In all experiments, n equals the number of subjects, and results are expressed as median value or mean ± SD. For comparison of unpaired and not normally distributed data, the Mann-Whitey test was used. For paired, not normally distributed data, Wilcoxon matched-pairs signed-rank test was used. We used a type 1 error rate α level of 0.05 and a 2-sided test. The data were analyzed with GraphPad Prism software (Version 6.0, San Diego, California).

## Results

### Notch expression on T-cells in nasal mucosa and blood

**Fig. 1:**
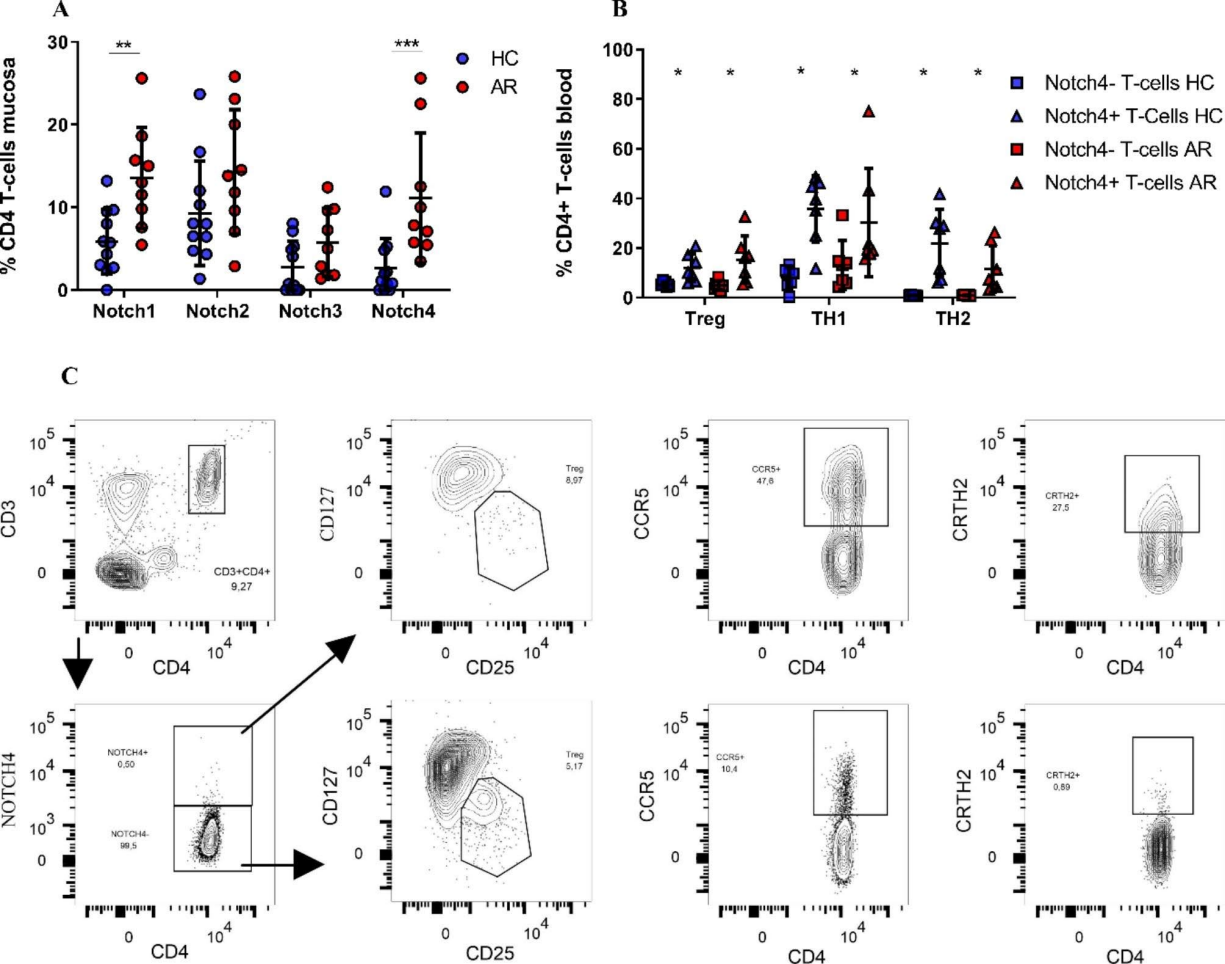
Notch1-4 on T-cells in nasal mucosa and blood. **A**: Fraction of CD4 T-cells in nasal mucosa expressing Notch. **B**: Distribution of CCR5^+^, CRTH2^+^ and CD25^++^CD127^low^ within blood-derived Notch-4^+^ respectively Notch-4^−^ CD4^+^ T-cells. **C**: Visualization of the flow cytometry analysis of Th1, Th2, and Treg from one healthy control patient. Allergic rhinitis (AR), healthy controls (HC). Horizontal lines represent the mean value and SD. p < 0.05=*, p < 0.01= **, p < 0.001= **

Expression of Notch1-4 on T-cells was assessed by flow cytometry. The fraction of CD4^+^ T-cells expressing Notch1 and Notch4 in nasal mucosa was significantly higher in AR patients compared to Healthy control patients (AR: 13.58%±6.1%; 11.1 ± 7.8%, n = 9, HC: 3.9%±2.0%; 2.6 ± 3.6%, n = 11, Notch1 p < 0.01, Notch4 p < 0.001) (Fig. 1A). Flow cytometry data is supplemented (Supplementary Fig. 1A, B). No differences in Notch expression were detected in blood-derived CD4^+^ T-cells (Supplementary Fig. 2). In CD4^+^Notch4^+^ blood-derived T-cells, significantly higher levels of CCR5^+^ (T_h_1), CRTH2^+^ (T_h_2), and CD25^++^CD127l^ow^ (T_reg_ cells) were found compared to CD4^+^ Notch4^−^ blood derived T-cells both in Healthy control patients and AR patients (Fig. 1B). AR Notch4^−^: CCR5^+^ 13.2%±9.8%; CRTH2^+^ 1.0%±0.2%; CD25^++^CD127^low^ 5.0%±1.8%, AR Notch4^+^ CCR5^+^ 30.2%±21.8%; CRTH2^+^ 11.6%±9.4%; CD25^++^CD127^low^ 15.3%±9.5%, T_h_1 p < 0.05, T_h_2 p < 0.05, T_reg_ p < 0.05. Similar levels for CCR5^+^, CRTH2^+^, and CD25^++^CD127^low^ were detected in healthy control patients. Figure 1 C visualizes flow cytometry data for Notch4, T_h_1, T_h_2, and T_reg_.

### Notch ligand expression on epithelial cells in the nasal mucosa

**Fig. 2:**
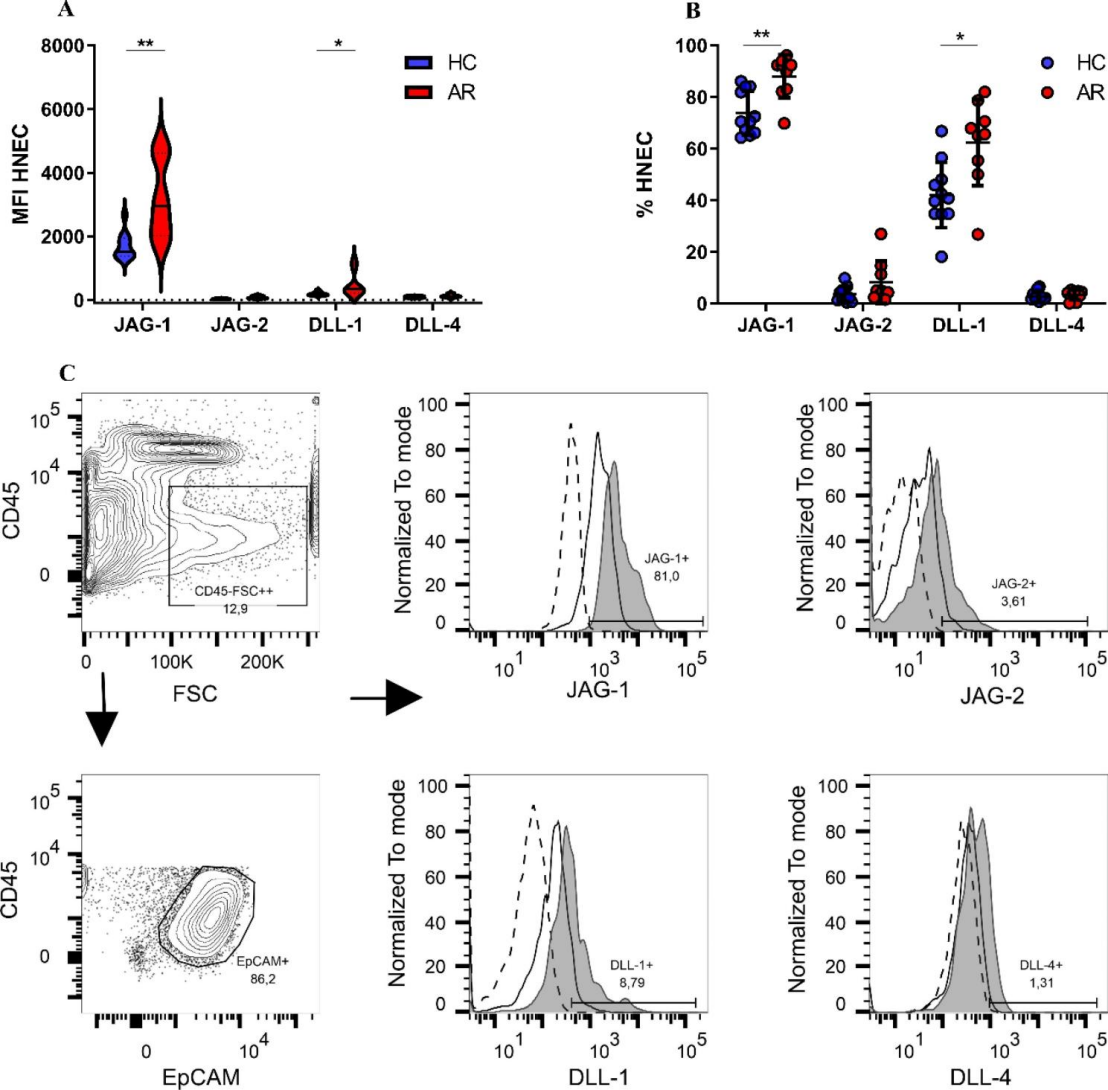
Expression of Notch ligands on epithelial cells in the nasal mucosa. **A**: In epithelial cells, the median fluorescence intensity of JAG-1, JAG-2, DLL-1, and DLL-4 are displayed with violin plots; the horizontal line represents the median value. **B**: Fraction of epithelial cells expressing Notch ligands, the horizontal lines represent the mean value and SD. **C**: Flow cytometry analysis of Notch ligands, data from one healthy control (HC) patient and one allergic rhinitis (AR) the patient is visualized. Dotted line = FMO control, black line = HC patient, Filled grey = AR. Patients with AR, (n = 9), HC, (n = 11). p < 0.05= *, p < 0.01= **

The expression of JAG-1, JAG-2, DLL-1, and DLL-4 were assessed with flow cytometry. JAG-1 and DLL-1 expression was generally higher than JAG-2 and DLL-4 expression. The expression of JAG-1 and DLL-1 on epithelial cells analyzed by median fluoresce intensity (MFI) was significantly elevated in AR patients compared to Healthy control patients (JAG-1 in AR: 3188 ± 1231, n = 9, HC: 1692 ± 418, n = 11, p < 0.001), (DLL-1 in AR: 472 ± 389, n = 9 and Healthy control: 191 ± 54, n = 11, p < 0.05) (Fig. 2A). No differences were detected in the expression of JAG-2 or DLL-4 on epithelial cells when comparing specimens from AR patients and healthy control patients. The increased expression of JAG-1 and DLL-1 among patients with AR could be further corroborated by analyzing the fraction of cells expressing JAG-1 or DLL-1. The fraction of epithelial cells expressing JAG-1 was significantly elevated in AR patients compared to Healthy control patients (AR: 88.05%±8.3%, n = 9, Healthy control patients: 73.9%±8.4%, n = 11, p < 0.01) (Fig. 2B). In addition, the fraction of DLL-1 expressing epithelial cells was also found to be significantly increased in AR patients compared to Healthy control patients (AR: 62.4 ± 16.7%, n = 9, Healthy control patients: 42.0 ± 12.7% n = 11, p < 0.01) (Fig. 2B). When comparing specimens from AR and healthy control patients, no differences were detected in the fraction of epithelial cells expressing JAG-2 or DLL-4. Figure 2 C visualizes the expression level for JAG-1, JAG-2, DLL-1, and DLL-4 in one healthy control patient and one AR patient.

### Notch ligand on neutrophils in nasal mucosa and blood

**Fig. 3:**
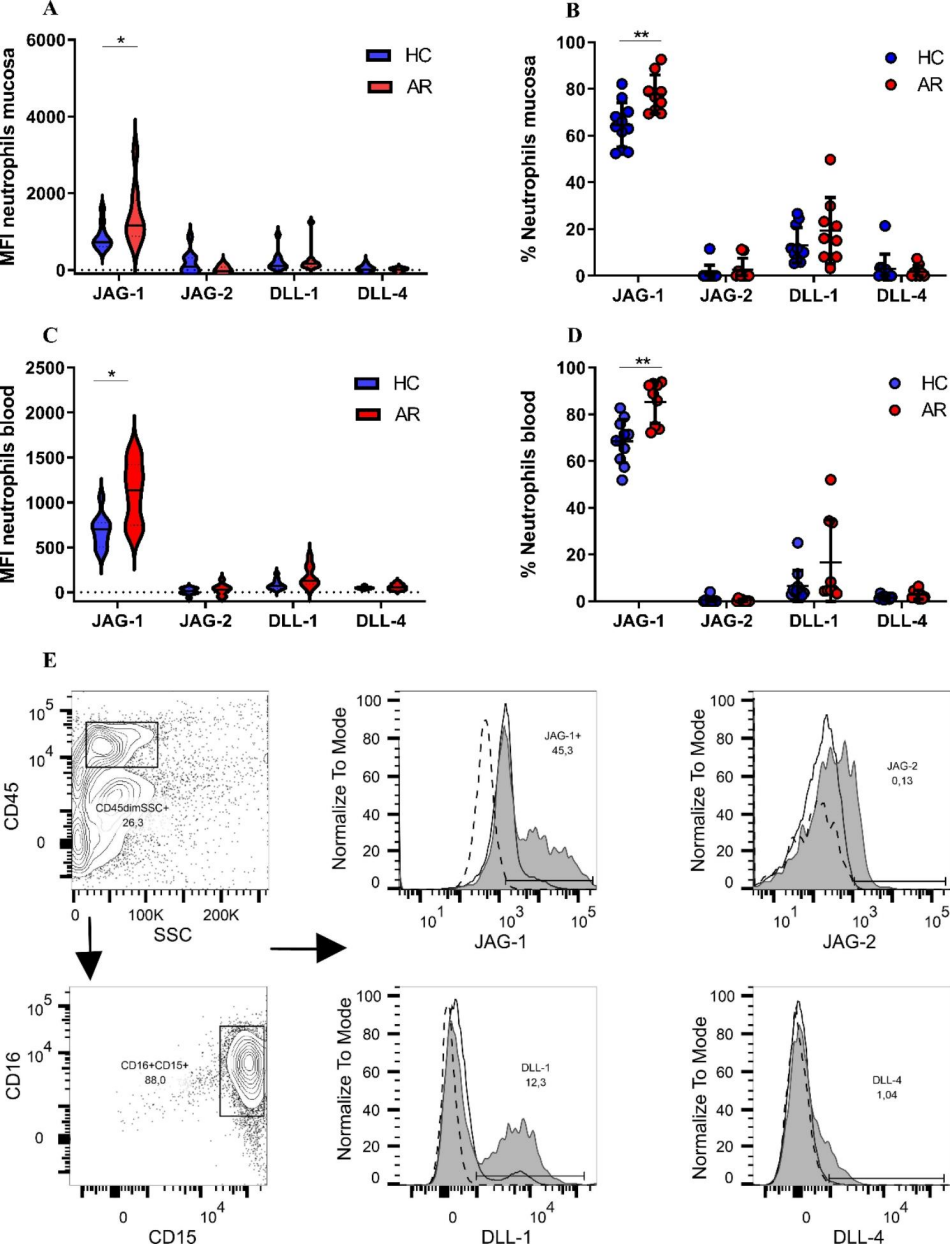
Notch ligands on neutrophils in nasal mucosa and blood. Median fluorescence intensity of JAG-1, JAG-2, DLL-1, and DLL-4 in **A**: Nasal mucosa-derived neutrophils and **B**: Blood-derived neutrophils are displayed with violin plots; the horizontal line represents the median value. Fraction of neutrophils expressing Notch ligands in **B**: Nasal mucosa and **D**: Blood, Horizontal lines represent the mean value and SD. **E**: Flow cytometry analysis of Notch ligands, data from one healthy control (HC) patient and one allergic rhinitis (AR) the patient is visualized. Dotted line = FMO control, black line = HC, Filled grey = AR. Patients with AR, (n = 9), HC, (n = 11). p < 0.05= *, p < 0.01= **

The expression of JAG-1, JAG-2, DLL-1, and DLL-4 on neutrophils was assessed with flow cytometry. Expression measured by MFI of JAG-1 on neutrophils was significantly increased in AR patients compared to healthy control patients in samples derived from nasal mucosa and blood. (JAG-1 MFI on mucosal neutrophils from AR: 1405 ± 758, n = 9 vs. Healthy control patients: 855 ± 328, n = 11, p < 0.05) (Fig. 3A). (JAG-1 MFI on blood-derived neutrophils in AR: 1110 ± 343, n = 9 vs. Healthy control patients: 676 ± 181, n = 11, p < 0.05) (Fig. 3C). No differences between AR patients and healthy control patients were detected for the neutrophil expression of JAG-2, DLL-1, and DLL-4. In addition, the fraction of neutrophils expressing JAG-1 was significantly increased in AR patients compared to healthy control patients, in samples derived from both nasal mucosa and blood ( mucosa AR: 77.7%±8.3%, n = 9, mucosa Healthy control patients: 64.6%±9.5%, n = 11, p < 0.01) (blood AR: 85.3%±9.1%, n = 9, blood Healthy control patients: 68.5%±9.2%, n = 11, p < 0.01) (Fig. 3B, D). Figure 3E visualizes the expression level for JAG-1, JAG-2, DLL-1, and DLL-4 in one healthy control patient and one AR patient.

## Discussion

The present study demonstrates that outside pollen season in the nasal mucosa, the fraction of CD4^+^Notch1^+^ and CD4^+^Notch4^+^ T-cells is increased in AR patients. Correspondingly, AR patients displayed an increased expression level of Notch ligands on nasal epithelial cells and neutrophils. In AR patients, the expression of JAG-1 was one-fold higher, and DLL-1 were around 1.5-fold higher compared to the healthy controls. Also, neutrophils in the nasal mucosa of AR patients displayed a 75% increased expression of JAG-1 compared to neutrophils in healthy controls. The increased expression of JAG-1 and DLL-1 supports the notion that Notch interaction between CD4^+^ T-cells and nasal epithelial cells, respectively neutrophils, may regulate T-cells locally in peripheral tissue in AR patients. This increased expression of Notch ligands outside pollen season may be due to low-grade chronic allergic inflammation in nasal mucosa that prime the immune system upon allergen re-exposure.

Together with our previous report of nasal epithelial cells interacting with CD4^+^ T-cells [24], the present data suggest that increased JAG-1 and DLL-1 in allergic mucosa may promote a T-cell response favorable for the progression of AR inflammation. In analogy, neutrophils in AR mucosa might regulate T-cells expressing Notch receptors by their increased expression of JAG-1. This could be a potent regulator of T-cell function since neutrophils are one of the most abundant leukocytes in the allergic nasal mucosa [25]. Besides regulating T-cells, JAG-1 activation of epithelial cells has been shown to sustain the expression of IL-33, an interleukin that promotes a Th2 cell response [26]. The increased expression of JAG-1 detected in AR mucosa promotes allergic inflammation by maintaining IL-33 expression in nasal epithelial cells.

The present study also shows an increased fraction of CD4^+^ T-cells expressing notch1 and 4 in the nasal mucosa of AR patients. Activation of the Notch1 signalling pathway has been shown to promote the expression of T_h_2 genes. The increased fraction of CD4^+^Notch1^+^ T-cells seen in AR patients may reflect an increased presence of Th2 cells in the nasal mucosa. An alternative explanation is that the Notch1 expressing CD4^+^ T-cells is only a reflection of increased T-cell activation and not differentiation. Nevertheless, it is not inconceivable to think that the increased fraction of T-cells expressing Notch1 receptors in the nasal mucosa in AR patients reflects an immune system primed to respond to external pathogens. AR patients also displayed an increased fraction of CD4^+^ T-cells expressing Notch4 in the nasal mucosa. In the present study, no marker for Treg differentiation was included to differentiate if the Nocth4 expressing CD4^+^ T-cells in nasal mucosa were Tregs. This would have been interesting since activation of Notch4 signalling by JAG-1 in CD4^+^ Tregs has been shown to reduce their capacity to regulate airway inflammation [27]. In blood, Notch4^+^ and Notch4^−^ CD4^+^ T-cells were analyzed to detect the presence of differentiated Th cells within the population. Our data could confirm that Notch4^+^ CD4^+^ T-cells encompass an increased fraction of Th1, Th2, and Treg cells compared to Notch4^−^ T-cells. The same distribution was detected in both AR patients and healthy control patients. Furthermore, no difference in the expression of Notch receptors was seen on CD4^+^ T-cells in peripheral blood, comparing the levels between AR patients and the healthy control patients. It is essential to note that this study was performed outside the pollen season, and it cannot be excluded that an increase in Notch expressing CD4^+^ T-cells also can be seen in the blood of patients with severe pollen-induced symptoms.

In the present study, we could also show that neutrophils in AR patients displayed increased expression of JAG-1 in peripheral blood. The contribution of this finding to AR inflammation is unclear. However, neutrophils may be subjected to a systemic stimulus preparing them to express JAG-1 already in the blood. This hypothesis is based on the findings that high levels of short fatty acids in mice affect the maturation of dendritic in the bone marrow and, subsequently, the effector function in blood and peripheral tissue [28]. The lack of functional experiments with mediators that selectively block Notch1, 4, JAG-1, and DLL-1 mediated signalling in AR patients limits the study. Still, the descriptive data presented are novel in humans and corroborate previous animal assessments.

In summary, we have previously demonstrated that neutrophils and epithelial cells promote allergic mucosal inflammation by interacting with T-cells [24, 29]. Animal studies have indicated that the Notch pathway might be another way for these cells to promote Th2-induced upper airway allergic disease. The present study is the first to demonstrate an increased fraction of Notch1 and Notch4 expressing CD4^+^ T-cells in AR nasal mucosa and increased levels of JAG-1 and DLL-1 in mucosal epithelial cells, respectively JAG-1 on mucosal and blood-derived neutrophils. Together this signals an increased activity in the Notch1/4 - JAG-1/DLL-1 pathways among allergic individuals. Thus, propagating the importance of Notch signalling in AR and blocking JAG-1 and DLL-1-induced Notch signalling by nasal epithelial cells and Neutrophils are potential targets to reduce allergic airway inflammation.

## Electronic supplementary material

Below is the link to the electronic supplementary material.

Supplementary Figures and Tables

## Data Availability

The datasets during and/or analyzed during the current study are available from the corresponding author upon reasonable request.
