# Supplementary material for: Upregulated expression of Notch1/4 - JAG-1/DLL-1 detected in allergic rhinitis
Source: Allergy Asthma Clin Immunol. 2023 May 14;19:41. doi: 10.1186/s13223-023-00793-4 (PMC10183115; doi:10.1186/s13223-023-00793-4)
Supplement: Supplementary file 1 — Supplementary Figures and Tables [file 13223_2023_793_MOESM1_ESM.docx]

Supplement

**Methods**

Table S1 Patient characteristics


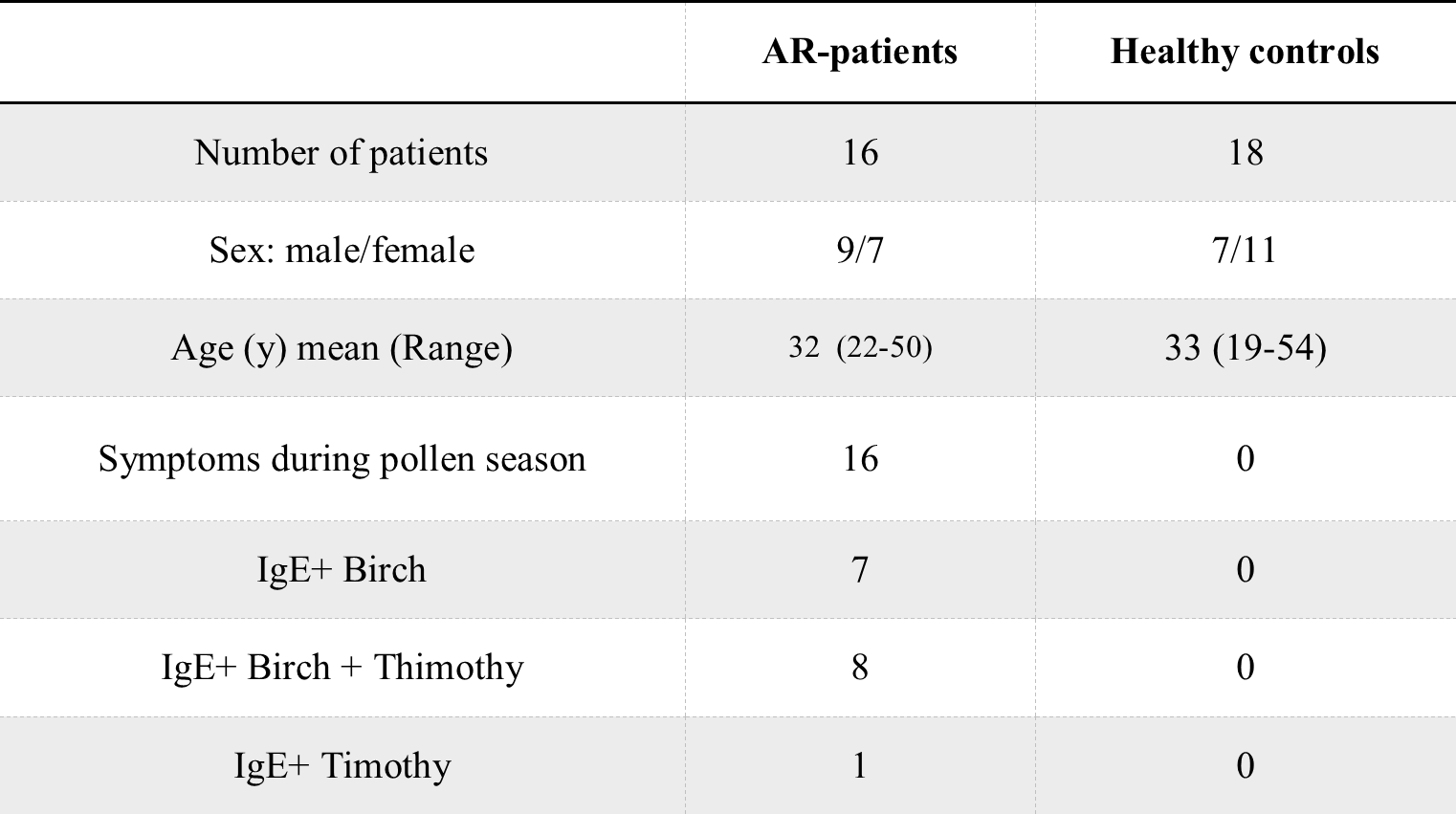


Table S2 Antibody panels


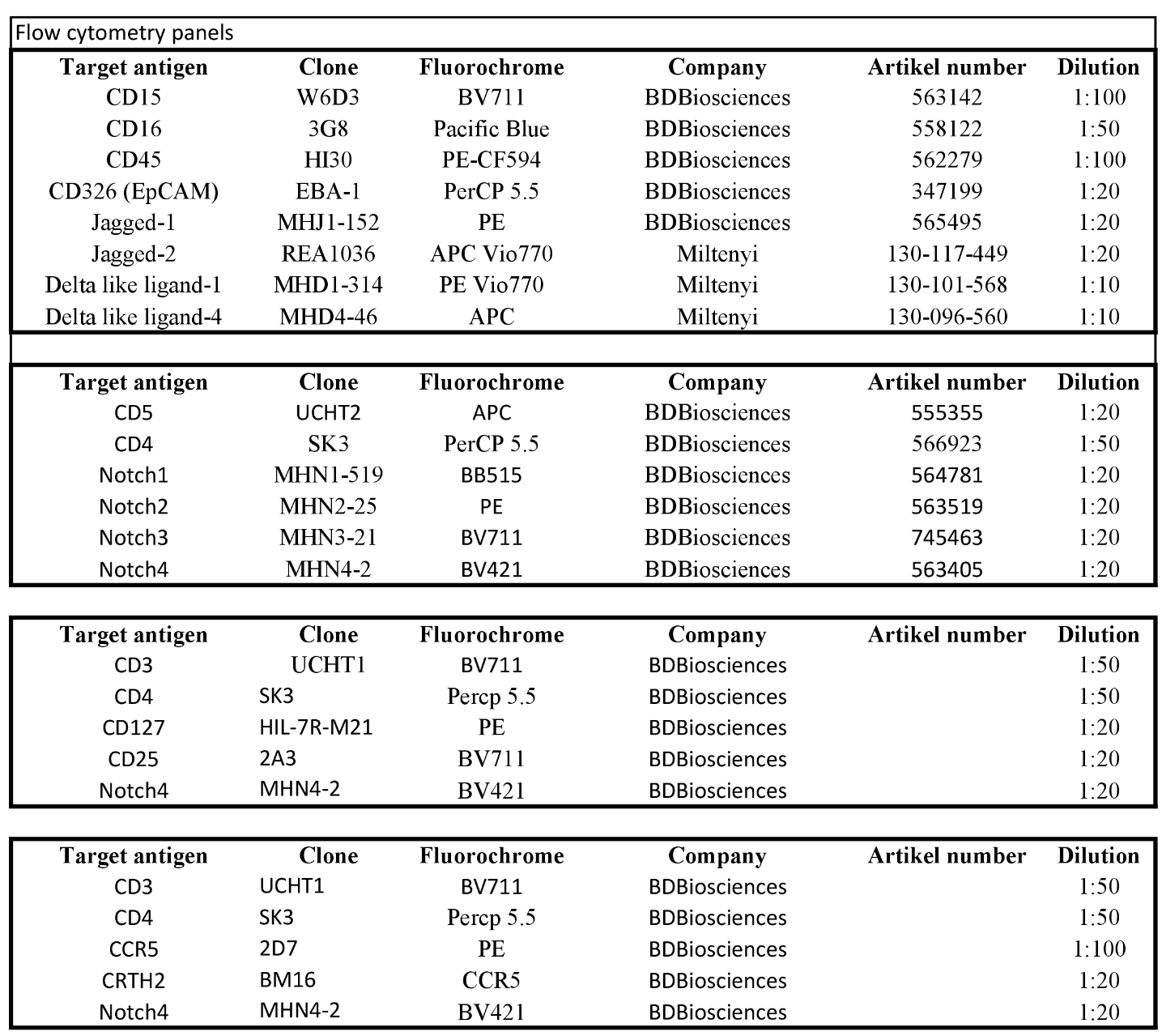


Results

Supplementary Figure 1A

Staining of CD4^+^ T-cells in the nasal mucosa


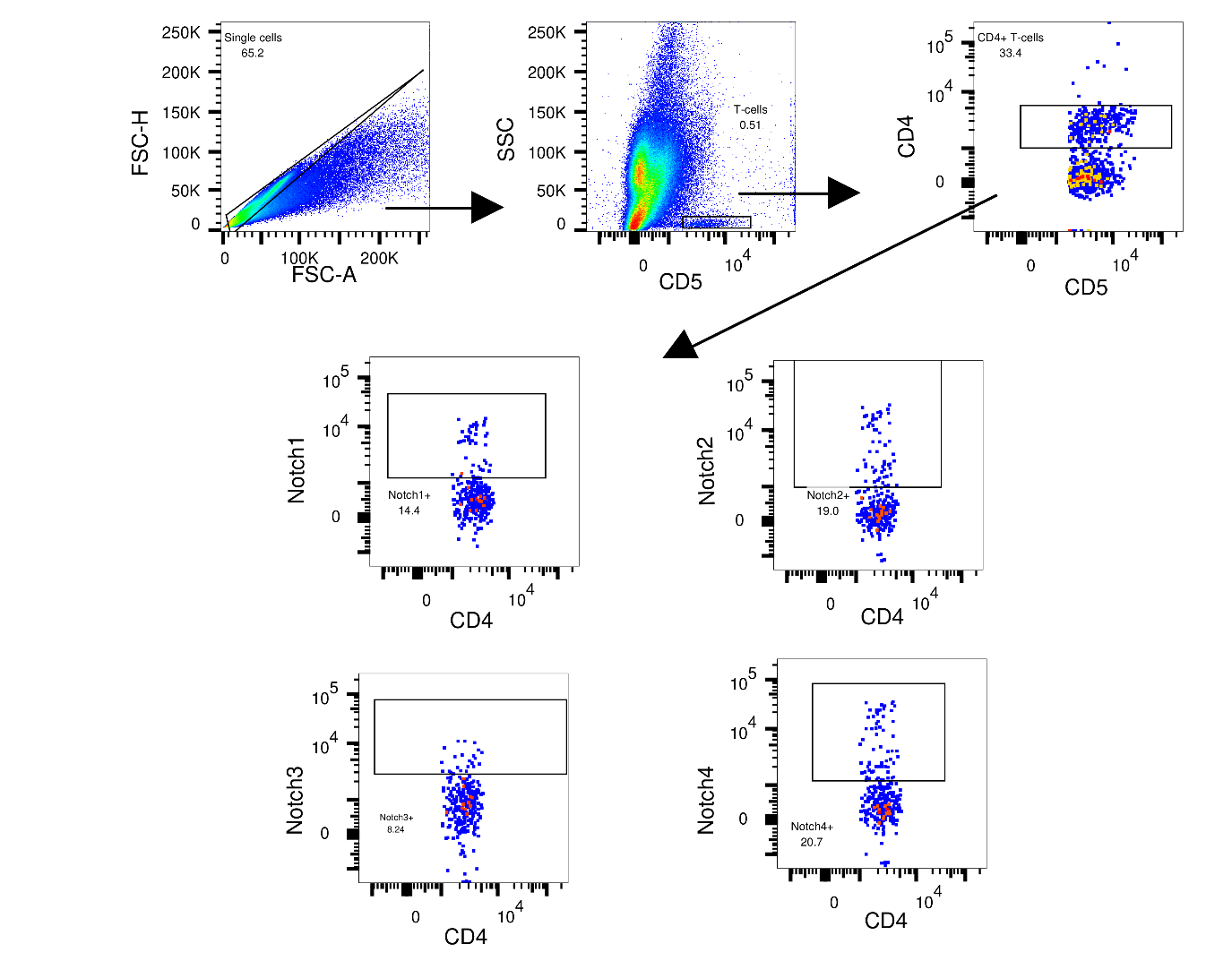


Supplementary figure A1. Gating of CD4+ T-cells in the nasal mucosa. First, single cells are gated with FSC-H and FSC-A. Next T-cells are gated with SSC vs. CD5. Subsequently, notch expression is analyzed with Notch vs CD4.

Supplementary Figure 1B

FMO control staining of CD4^+^ T-cells in the nasal mucosa


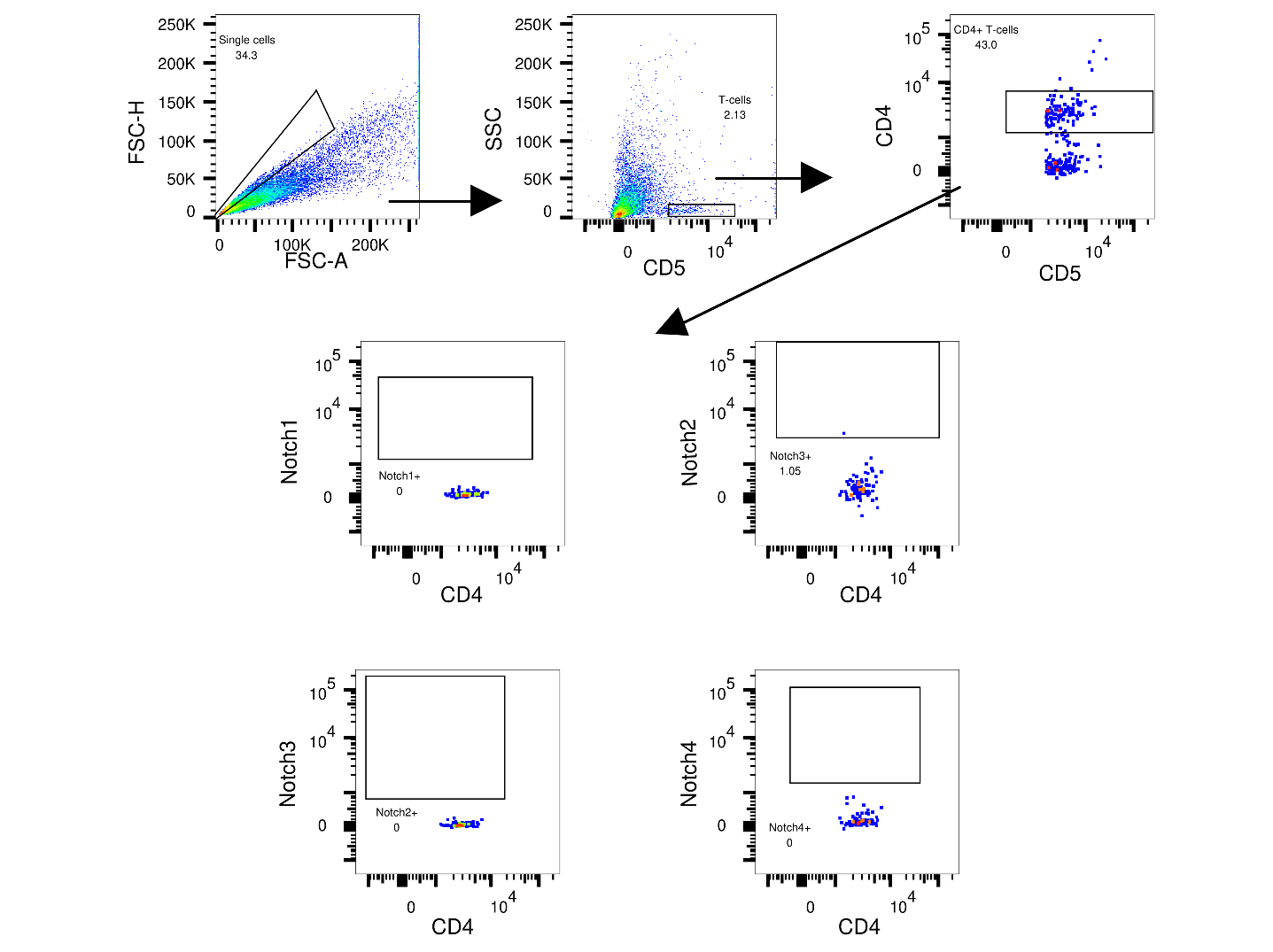


Supplementary figure A1. Gating of CD4+ T-cells in the nasal mucosa. First, single cells are gated with FSC-H and FSC-A. Next T-cells are gated with SSC vs. CD5. Subsequently, notch expression is analyzed with Notch vs. CD4.


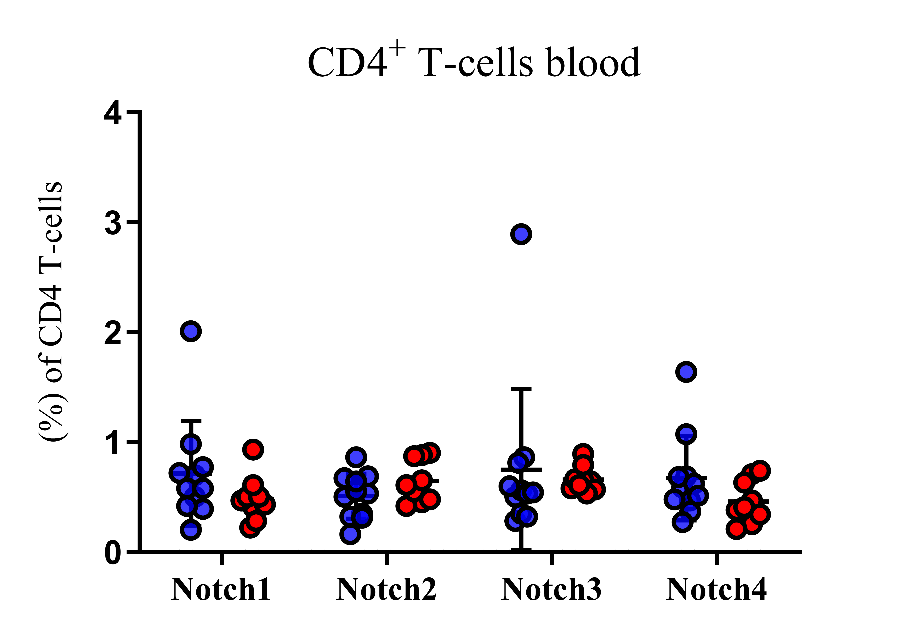


**Supplementary Figure S2 Notch expression in blood-derived CD4^+^ T-cells**

Fraction of Notch expression on CD4^+^ T-cells in peripheral blood. Healthy control patients (HC) and Allergic rhinitis patients (AR). HC n=11, AR n=9.
